# Supplementary material for: Netcombin: An algorithm for constructing optimal phylogenetic network from rooted triplets
Source: PLoS One. 2020 Sep 18;15(9):e0227842. doi: 10.1371/journal.pone.0227842 (PMC7500971; doi:10.1371/journal.pone.0227842)
Supplement: S4 File — (ZIP) [file pone.0227842.s004.zip › SequenceData-Triplet/sequenceData-10-20-30-0-Triplet/length 200, numseq 30, rate 10^-7/7 seq.pdf]

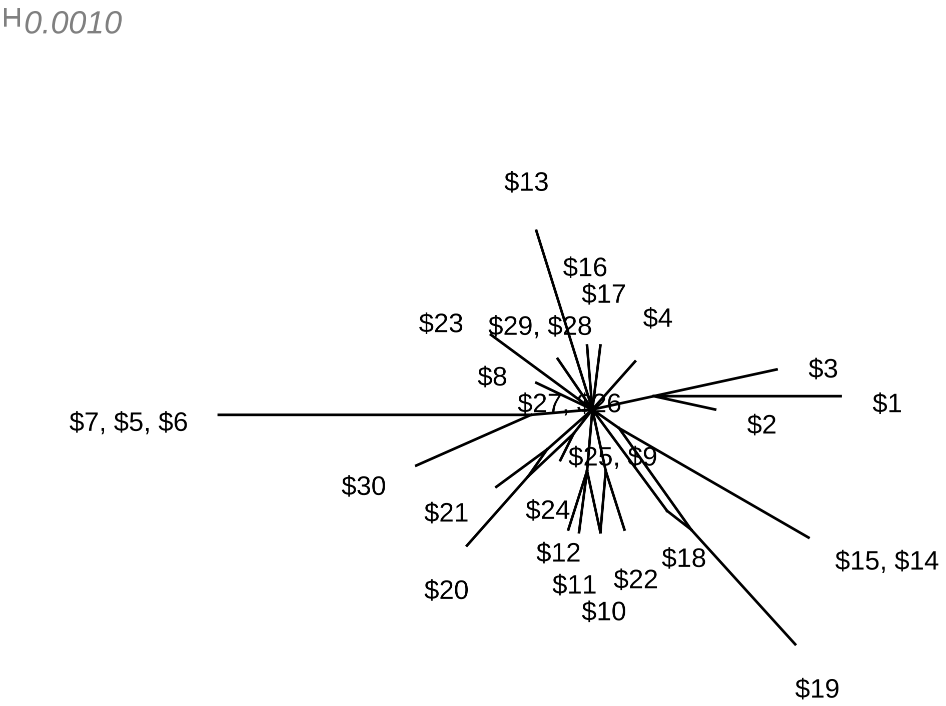
0.0010
$13
$16
$17
$23
$29, $28
$4
$3
$8
$27, $26
$1
$7, $5, $6
$2
$25, $9
$30
$21
$24
$12
$18
$15, $14
$22
$11
$20
$10
$19
